# Reconstruction and evaluation of oil-degrading consortia isolated from sediments of hydrothermal vents in the South Mid-Atlantic Ridge

**DOI:** 10.1038/s41598-021-80991-5

**Published:** 2021-01-14

**Authors:** Meng Ma, Li Zheng, Xiaofei Yin, Wei Gao, Bin Han, Qian Li, Aimei Zhu, Hao Chen, Huanghao Yang

**Affiliations:** 1 College of Biological Science and Engineering, Fuzhou University, Fuzhou, 350108 China; 2 Key Laboratory of Marine Eco-Environmental Science and Technology, First Institute of Oceanography, Ministry of Natural Resources, Qingdao, 266061 China; 3 Laboratory for Marine Ecology and Environmental Science, Pilot National Laboratory for Marine Science and Technology (Qingdao), Qingdao, 266071 China

**Keywords:** Ecology, Environmental sciences, Ocean sciences

## Abstract

In this study, sediments were collected from two different sites in the deep-sea hydrothermal region of the South Atlantic Ocean. Two microbial enrichment cultures (H7S and H11S), which were enriched from the sediments collected at two sample sites, could effectively degrade petroleum hydrocarbons. The bacterial diversity was analyzed by high-throughput sequencing method. The petroleum degradation ability were evaluated by gas chromatography–mass spectrometry and gravimetric analysis. We found that the dominant oil-degrading bacteria of enrichment cultures from the deep-sea hydrothermal area belonged to the genera *Pseudomonas*, *Nitratireductor*, *Acinetobacter*, and *Brevundimonas*. After a 14-day degradation experiment, the enrichment culture H11S, which was obtained near a hydrothermal vent, exhibited a higher degradation efficiency for alkanes (95%) and polycyclic aromatic hydrocarbons (88%) than the enrichment culture H7S. Interestingly, pristane and phytane as biomarkers were degraded up to 90% and 91% respectively by the enrichment culture H11S, and six culturable oil-degrading bacterial strains were isolated. *Acinetobacter junii* strain H11S-25, *Nitratireductor* sp. strain H11S-31 and *Pseudomonas* sp. strain H11S-28 were used at a density ratio of 95:4:1 to construct high-efficiency oil-degrading consortium H. After a three-day biodegradation experiment, consortium H showed high degradation efficiencies of 74.2% and 65.7% for total alkanes and PAHs, respectively. The degradation efficiency of biomarkers such as pristane and high-molecular-weight polycyclic aromatic hydrocarbons (such as CHR) reached 84.5% and 80.48%, respectively. The findings of this study indicate that the microorganisms in the deep-sea hydrothermal area are potential resources for degrading petroleum hydrocarbons. Consortium H, which was artificially constructed, showed a highly efficient oil-degrading capacity and has significant application prospects in oil pollution bioremediation.

## Introduction

Oil spills caused by human activities such as offshore oil exploration and marine oil transportation result in serious damage to the marine ecological environment^1^. Currently, the traditional approaches for removing marine spilled oil involve physical and chemical methods. Physical methods using oil booms and skimmers can quickly remove oil slicks from the sea surface but consume considerable labour power as well as material and financial resources. Chemical methods, including spraying dispersants or oil coagulants, will cause transformation of oil pollutants in applications and produce secondary pollution. Therefore, physical and chemical methods are often used in the early stage of marine oil spills cleanup^2,3^. Biodegradation is recognized as a natural process for eliminating petroleum hydrocarbon pollutants from the aquatic environment. Therefore, the development of efficient bioremediation technology is a promising and environmentally friendly approach for removing spilled oil from ocean water. In this way, obtaining microbial resources with efficient oil-degrading capability becomes the key issue for achieving bioremediation.

Natural hydrocarbons are abundant in deep-sea hydrothermal areas and can be used as a potential carbon source by specific microbial communities in these areas^4^. The biodegradation process for hydrocarbons in such areas has also been described^5^. Due to the extreme environmental characteristics of high temperature, high pressure, low oxygen concentration, and high heavy metal concentration, microorganisms have evolved unique physiological functions to adapt to extreme environments. Studies have reported that the metabolism of this microbial community is different from those of terrestrial and offshore microorganisms. The microorganisms in the deep-sea hydrothermal areas are considered to be a potential resource for petroleum degradation^6–8^.

Investigations have shown that the enrichment culture with oil as the sole carbon source can be used to obtain consortia with stable ability to degrade oil. Compared with single strains, microbial consortia composed of multiple strains exhibit better oil degradation efficiency. On one hand, this may be due to functional complementation among different species, leading to an increase in environmental adaptation and the reproduction of oil degraders in the community depending on the activities of other bacteria^9–12^. On the other hand, a variety of bacteria within a consortium contain more diverse set of metabolic pathways and a wider range of enzymes, expanding the metabolic range of hydrocarbon degradation. The synergistic effect of a bacterial consortium enhances the oil-degradation ability compared to a single oil degrader^13^. When the members of a successful bacterial consortium are highly dependent on each other, they form a more stable relationship with each other and maintain cell growth and perform functions more effectively. An increasing number of researchers have focused on constructing oil-degrading consortia to solve the problem of low degradation efficiency by an individual strain^1^. However, the bottleneck in constructing microbial consortia with great application potential is the lack of a reasonable design for the set of members in a consortium^14^.

Previous studies have isolated bacterial strains from soil contaminated with petroleum hydrocarbons and used these strains to establish oil-degrading consortia based on the survival of strains and their ability to degrade hydrocarbons^1,15^. However, these consortia still present certain challenges, such as complex construction procedures and unsatisfactory degradation effects. We think that effective oil-degrading consortium should be mimicking a natural community, minimizing the bacterial members with high oil degradation efficiency and reducing the complexity of the consortium^16^. Therefore, it is crucial to find a reliable strategy for reducing diversity to construct the best simplified consortium. In this study, we isolated high-efficiency oil-degrading bacterial strains from sediments collected from the deep-sea hydrothermal area of the South Mid-Atlantic Ridge. Based on the consortium design principles of interdependent and stable microbial interactions^17–20^, as well as the diversity abundance ratio obtained after three enrichment culture experiments, we selected the dominant culturable strains to construct the consortium. Then, the oil degradation effect of the consortium was investigated with the expectation of applying it to oil pollution bioremediation.

## Materials and methods

### Sampling information and medium

We collected two sediments from hydrothermal vents in the South Mid-Atlantic Ridge through the marine research vessel “Xiang-Yang-Hong 01” during the 46th cruise in 2017. The ambient temperature of sediments was about 2.6 °C. We wrapped the sediment samples by tin foil and stored them at 4 °C. The ambient environmental parameters are listed in Online Resource Table S1.

The media used in this study included organic medium M8^21^, basic medium ONR7a, and oil medium (100 mL ONR7a medium, of which 1% (w/v) crude oil was the sole source of carbon and energy^22^). All media were sterilized at 121 °C for 15 min.

### Isolation and screening in crude oil degraders

Five grams of sediment from each of two different samples was added to 100 mL of oil medium. Oil medium with no sediment was used as a negative control. Each treatment was tested in triplicate and incubated at 25 °C with shaking at 115 rpm for 14 days under aerobic conditions. Then, 2 mL of the enriched cultures was transferred to fresh oil medium and incubated under the same conditions. The oil enrichments have been transferred three times. Then, the enrichment cultures were serially diluted and plated on M8 agar plates and incubated at 25 °C for 4 days. The bacterial colonies with different morphologies were picked and streaked on fresh M8 plates for further purification. All isolated strains were stored in M8 medium containing 30% glycerol at − 80 °C for further analysis.

### Phylogenetic analysis of the isolates

The 16S rRNA gene sequences of purified strains were amplified with 27F and 1492R sequencing primers by a thermal circulator (Takara, Japan). The purified PCR products were sequenced by TSINGKE company (TSINGKE, Qingdao, Shandong province, China). Next, the obtained partial 16S rRNA gene sequences were uploaded to the GenBank nucleotide sequence database and aligned using BLAST. The accession numbers are shown in Table 1. The neighbour-joining method in MEGA software (version 7.0, https://www.megasoftware.net/mega4/) was utilized to reconstruct the phylogenetic tree.

**Table 1:** The oil-degrading bacteria isolated from the enrichment consortium H11S and the corresponding type strains.

Strains (GenBank accession no.)	Most closely related type strain	Similarity/%
H11S-25 (MK680493)	*Acinetobacter junii* strain 65^T^ (CP019041.1)	99.93
H11S-28 (MK729061)	*Pseudomonas* sp. strain BS^T^ (MH915623.1)	99.00
H11S-31 (MK729062)	*Nitratireductor* sp. strain 7002-098^T^ (KY770526.1)	99.93
H11S-32 (MK729063)	*Microbacterium lacticum* strain 3388^T^ (EU714364.1)	99.36
H11S-35 (MK729064)	*Nitratireductor* sp. strain 7002-098^T^ (KY770526.1)	99.93
H11S-71 (MK729065)	*Erythrobacter* sp. strain JLT71^T^ (KX989323.1)	99.93

### Oil degradation ability of the isolated strains

Seed solution (200 μL) of different oil-degrading strains related less than 100% based on their 16S rRNA genes phylogenetic analysis was transferred to 50 mL fresh oil medium. A 50 mL volume of oil medium without bacteria was used as a negative control. All experimental treatments were cultured under aerobic conditions in duplicate for 14 days at 25 °C with shaking at 150 rpm. Degradation efficiency was tested by gravimetric and GC–MS analysis^23^.

After 14 days of degradation, the residual oil was extracted with 50 mL dichloromethane. As described previously^24^, 20 mL of the organic phase was accurately collected and dried under stream of nitrogen to determine the crude oil degradation ability via gravimetric analysis. The oil degradation efficiency for both test and control groups (D) was calculated by the following formula:

$$D = \frac{M0 - 2.5M}{{M0}} \times \, 100\%$$

In the equation, M_0_ is the weight of oil initially added to the medium. M is the weight of residual oil after extraction.

The oil degradation efficiency of the strains or consortia (D_E_) was evaluated by the following formula:

$${\text{D}}_{{\text{E}}} = {\text{ D}}_{{\text{T}}} - {\text{ D}}_{{\text{C}}}$$

In the equation, D_T_ is the oil degradation efficiency of the test group. D_C_ is the oil degradation efficiency of the control group.

For analysis of total hydrocarbon and PAHs degradation, 2 mL of residual organic phase was dehydrated with 2 g of anhydrous Na_2_SO_4_ and filtered through a 0.22 μm nylon membrane (JINTENG, Tianjin, China). The filtrate was evaporated under nitrogen flow at 25 °C and immediately dissolved in 1 mL chromatographic n-hexane. N-tetracosane-D50 and P-terphenyl-D14 (10 μg·mL^−1^ each) were added as internal standards. A 6890A gas chromatograph (Agilent Technologies) with an HP-5 MS capillary column (30 m × 250 μm i.d., 0.22 μm thickness) connected to a 5973 mass spectrometer equipped with a quadrupole axis detector was utilized to analyse the components. Degradation efficiencies of alkanes and PAHs were also calculated by the following formula:

$$DPAHs = \frac{{\sum {\text{PAHs-control}} - \sum {PAHs} }}{{\sum {\text{PAHs-control}} }} \, \times \, 100\%$$

$$D{\text{alkanes}} = \frac{{\sum {\text{alkanes-control}} - \sum {{\text{alkanes}}} }}{{\sum {\text{alkanes-control}} }} \, \times \, 100\%$$

In the equation, D_PAHs_ and D_alkanes_ are the degradation efficiencies of PAHs and *n*-alkanes respectively. ∑_PAHs-control,_ ∑_alkanes-control_, ∑_PAHs_ and ∑_alkanes_ are the PAHs and *n*-alkane residual concentrations in the crude oil of the control and test groups after biodegradation respectively.

### Microbial diversity analysis

The total genomic DNA of two enrichment cultures was extracted using the CTAB/SDS method, and the 16S rRNA genes of distinct regions (V3 + V4) were amplified used specific primer (341F-806R) with the barcode. All PCR reactions were carried out with Phusion High-Fidelity PCR Master Mix and the sequencer is Illumina MiSeq. The tag sequence was subjected to de-redundancy with the Mothur (v.1.34.0, http://www.mothur.org/) software package. Finally, the annotated sequences of the OTUs were obtained by assigning sequences with ≥ 97% similarity to the same OTUs. Alpha-diversity indices (ACE, Chao1, Shannon, and Simpson) were calculated with QIIME (Version 1.7.0, http://qiime.org/1.7.0/) and displayed with R software (Version 2.15.3; Team, R. C. R. A Language and Environment for Statistical Computing. Vienna, Austria. 2013, https://cran.r-project.org/) at a 97% similarity level.

### Construction and evaluation of oil-degrading consortia

A total of four consortia were constructed (consortia H, A21, B21 and C21). Based on the diversity of the enrichment culture H11S at the genus level, three strains including *Acinetobacter junii* strain H11S-25, *Nitratireductor* sp. strain H11S-31 and *Pseudomonas* sp. strain H11S-28 with the highest abundance in the final enrichment culture were selected to construct the consortia. These strains were transferred to an erlenmeyer flask containing 100 mL of M8 medium and cultivated at 25 °C until the OD_600_ value was equal to 0.3. Bacterial cultures constructed according to the scheme (Table 2), were centrifuged at 10,000 rpm for 10 min. The bacterial pellets were suspended in 1 mL ONR7a medium and then transferred to 100 mL of oil medium. The scheme of composite consortium H was based on the abundance ratio of the three strains in the enrichment culture H11S and formulated with an inoculation ratio for *Acinetobacter junii* strain H11S-25: *Nitratireductor* sp. strain H11S-31: *Pseudomonas* sp. strain H11S-28 of 95:4:1. The schemes of composite consortia A21, B21 and C21 were based on the abundance ratio of the two strains in the enrichment culture H11S. Consortium A21 was formulated with an inoculation ratio for *Acinetobacter junii* strain H11S-25: *Nitratireductor* sp. strain H11S-31 of 95:4. Consortium B21 was formulated with an inoculation ratio for *Nitratireductor* sp. strain H11S-31: *Pseudomonas* sp. strain H11S-28 of 4:1. Consortium C21 was formulated with an inoculation ratio for *Acinetobacter junii* strain H11S-25: *Pseudomonas* sp. strain H11S-28 of 95:1. Oil medium without bacteria was used as a negative control. Each treatment was tested in triplicate and incubated at 25 °C with shaking at 150 rpm for 3 days, 9 days, 15 days, and 21 days under aerobic conditions. The oil degradability of constructed consortium H was evaluated by comparing the oil degradation efficiency as measured by the gravimetric and GC–MS methods.

**Table 2:** The scheme of strain abundance ratio in constructed consortia.

Strains or consortia	Bacteria strains included	Abundance ratio	Degradation day (d)
H11S-25	*Acinetobacter junii* strain H11S-25	100	3、21
H11S-28	*Pseudomonas* sp. strain H11S-28	100	3、21
H11S-31	*Nitratireductor* sp. strain H11S-31	100	3、21
A21	*Acinetobacter junii* strain H11S-25: *Nitratireductor* sp. strain H11S-31	95:4	21
B21	*Nitratireductor* sp. strain H11S-31: *Pseudomonas* sp. strain H11S-28	4:1	21
C21	*Acinetobacter junii* strain H11S-25: *Pseudomonas* sp. strain H11S-28	95:1	21
H	*Acinetobacter juni*i strain H11S-25: *Nitratireductor* sp. strain H11S-31: *Pseudomonas* sp. strain H11S-28	95:4:1	21

## Results and discussion

### Biodegradability of crude oil by two enrichment cultures

**Figure 1:**
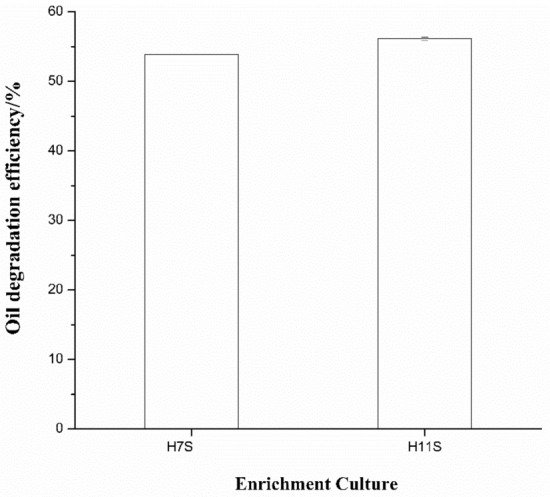
The oil degradation efficiency of the two enrichment cultures H7S and H11S.

The enrichment culture H7S showed no obvious proliferation in the first five days because sample 7S was a sulphide rock, while H11S showed visible proliferation after the fourth day. After 14 days of cultivation, gravimetric analysis demonstrated that the enrichment cultures H7S and H11S exhibited similar oil-degrading abilities and degraded 54% and 56% of the crude oil, respectively (Fig. 1).

**Figure 2:**
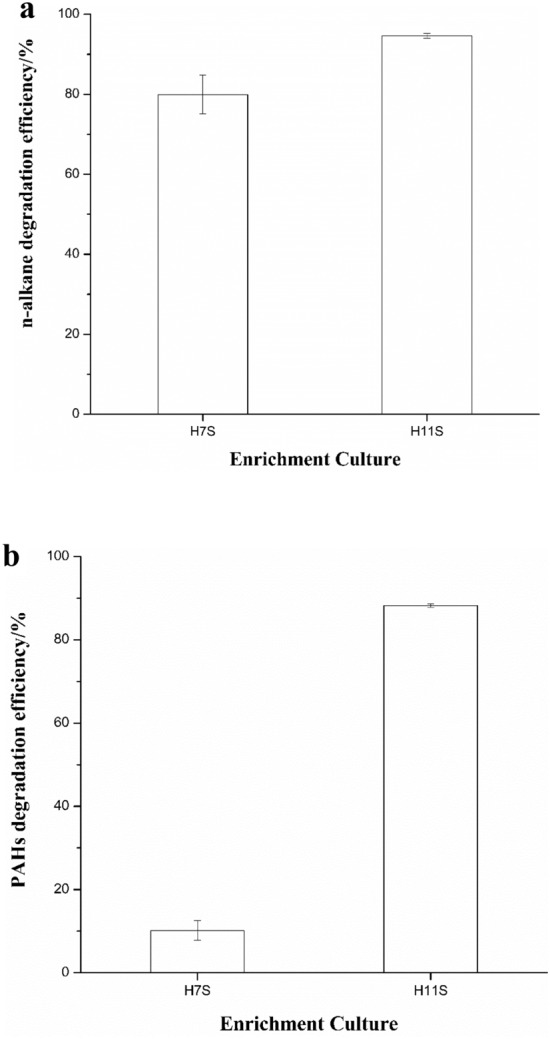
The total *n*-alkane and PAHs degradation efficiency of two enrichment cultures H7S and H11S (**a**) *n*-alkane degradation efficiency, (**b**) PAHs degradation efficiency.

The biodegradation percentages for total *n*-alkanes (C10–C34) and polycyclic aromatic hydrocarbons (PAHs) were calculated by comparing the two enrichment cultures with the negative controls (Fig. 2). Based on evaluation with C17/pristane and C18/phytane, the degradation efficiencies of the two enrichment cultures were significantly better than those of the negative controls (*P* < 0.01)^25^. In detail, the enrichment cultures H7S and H11S exhibited significant *n*-alkane degradation abilities, degrading 80% and 95% of the *n*-alkane components respectively. In addition, the enrichment culture H11S also exhibited a superior aromatic compound degradation efficiency (88%) which was much higher than that of the enrichment culture H7S (10%). Clearly, the efficiency of *n*-alkane removal was higher than that for PAHs, suggesting that the enrichment culture H7S was more inclined to biodegrade *n*-alkanes than PAHs.

**Figure 3:**
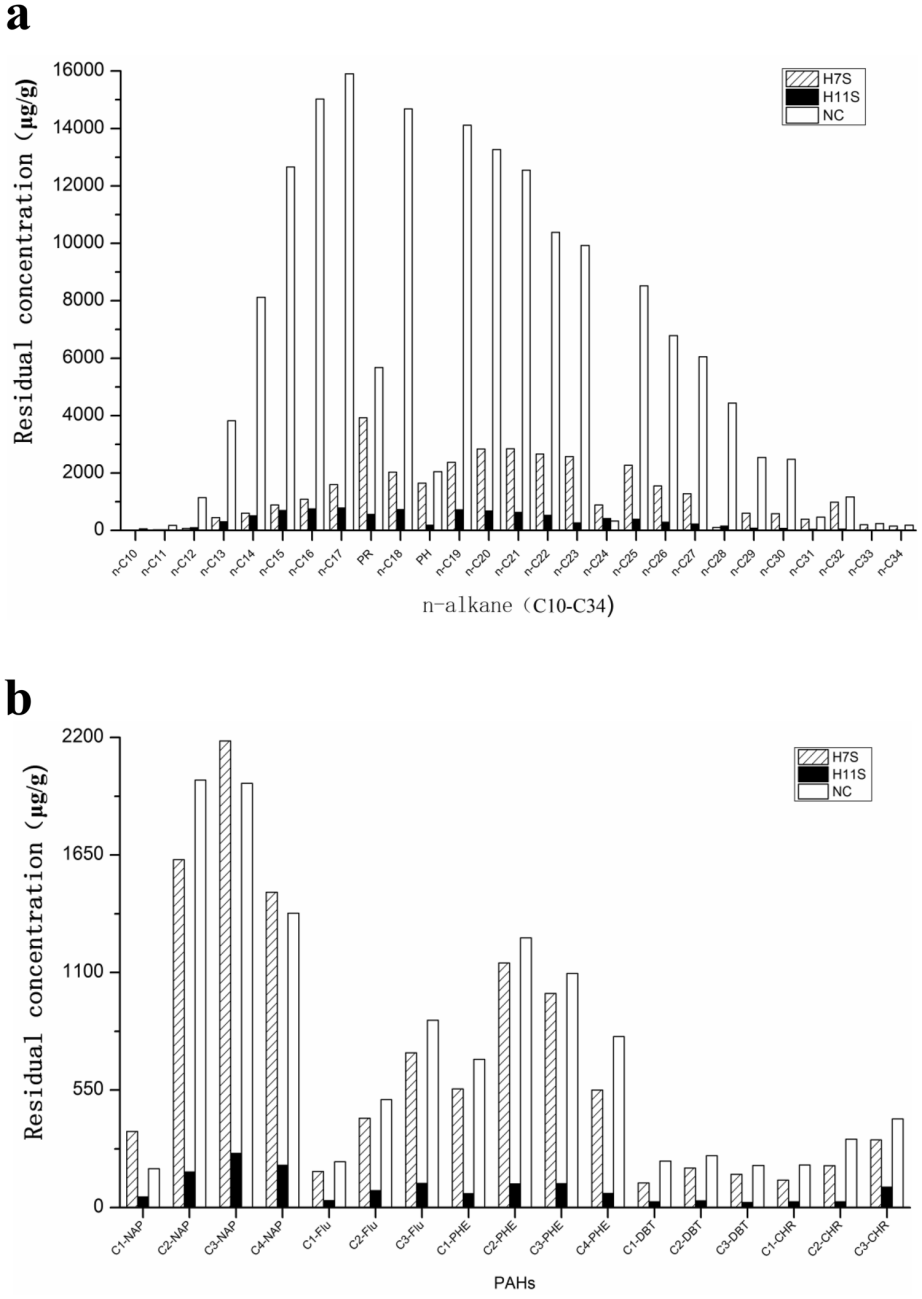
The n-alkanes and PAHs residual concentration of the crude oil after biodegradation by two enrichment cultures (**a**) *n*-Cm: alkane with m carbon atom, for instance, *n*-C16 is alkane with 16 carbon atom; "PR" is pristane, "PH" is phytane. (**b**) The concentrations of PAHs after the biodegradation by the 5 strains (NAP, FLU, DBT, PHE, and CHR represent for naphthalene, fluorine, dibenzothiophene, phenanthrene, chrysene, respectively. C1(C2, C3, C4)-NAP, alkylated naphthalene with straight-chain of 1 ~ 4 carbon atoms; C1(C2, C3)-FLU, alkylated fluorene with straight-chain of 1 ~ 3 carbon atoms; C1(C2, C3)-DBT, alkylated dibenantherene with straight-chain of 1 ~ 3 carbon atoms; C1(C2, C3, C4)-PHE, alkylated phenanthrene with straight-chain of 1 ~ 4 carbon atoms; C1(C2)-CHR, alkylated chrysene with straight-chain of 1 ~ 2 carbon atoms).

GC–MS analysis confirmed that each enrichment culture was able to use multiple oil components compared with the control group (Fig. 3a). The enrichment culture H7S was more active in degrading short-chain *n*-alkanes (C10–C19) with the degradation efficiency exceeding 80% and even reaching 93%. However, the ability to degrade long-chain alkanes was unsatisfactory, and the highest degradation efficiency was only 17%. In contrast, the enrichment culture H11S featured a broader degradation range and superior degradation capability. Degradation percentages exceeded 84% even reaching 97% for the *n*-alkanes within the entire range (C10-C34). It is worth noting that a particularly favourable degradation effect was observed for long-chain (C31–C34) *n*-alkanes with degradation efficiencies of approximately 91%, 95%, 92% and 92%, respectively. Interestingly, the biomarkers pristane (PR) and phytane (PH) were degraded by the enrichment culture H11S at high degradability levels of 90% and 91% respectively. PR and PH are commonly used to evaluate the biodegradability of oil or determine the source of an oil spill because they are difficult to degrade compared with *n*-alkanes^26^. The removal efficiencies for PR and PH by a previously reported bacterial consortium were only 40.1% and 39.5% respectively^27^. However, our results showing high biodegradability of PR and PH indicated that the enrichment culture H11S offered a superior potential for alkane degradation.

The residual concentration of PAHs in crude oil is shown in Fig. 3b. The enrichment culture H11S played a more active role in the biodegradation of alkylated PAHs (72%-92%) than did the enrichment culture H7S (less than 20%). In particular, the enrichment cultures H7S did not effectively degrade low-molecular-weight polycyclic aromatic hydrocarbons. However, it had excellent degradation effects on high-molecular-weight polycyclic aromatic hydrocarbons, such as alkylated dibenzothiophene (DBT) and alkylated chrysene (CHR). These degradation trends also indicate that increasing the ring number of PAHs, the enrichment culture exerts a positive effect on the biodegradation of the compounds^27^.

### Biodiversity and community structure of two enrichment cultures

QIIME software (version 1.9.1) was used to compare and analyse the alpha diversity of the two enrichment cultures. A total of 81,780 and 90,755 reads were obtained via Illumina MiSeq sequencing of the 16S rRNA gene sequences of the two enrichment cultures H7S and H11S, respectively. At least 97.4% of effective readings were obtained after screening, and the optimized read data showed corresponding lengths of 420 bp and 407 bp, respectively.

The rank abundance curve (Online Resource Fig. S1) indicated that the species richness of the enrichment culture H7S was significantly higher than that of the enrichment culture H11S. In the vertical direction, the smoothness of the curve for the enrichment culture H7S reflected that the species distribution was more uniform. According to statistics, 112 OTUs were gathered from two consortia, and the OTU content of the enrichment culture H7S was much higher than that of the enrichment culture H11S. The Venn diagram of two enrichment cultures H7S and H11S showed that 35 OTUs were shared and exhibited 31% similarity via the RDP Classifier (Online Resource Fig. S2). In addition, the community richness (ACE and Chao) and diversity (Shannon and Simpson) indices of the enrichment culture H7S were higher than those of H11S. The comparison of the Shannon diversity index showed that the microbial community of the enrichment culture H7S was composed of a greater number of phylotypes. The Simpson index also indicated that the favourable characteristics of the enrichment culture H7S were more conspicuous.

**Figure 4:**
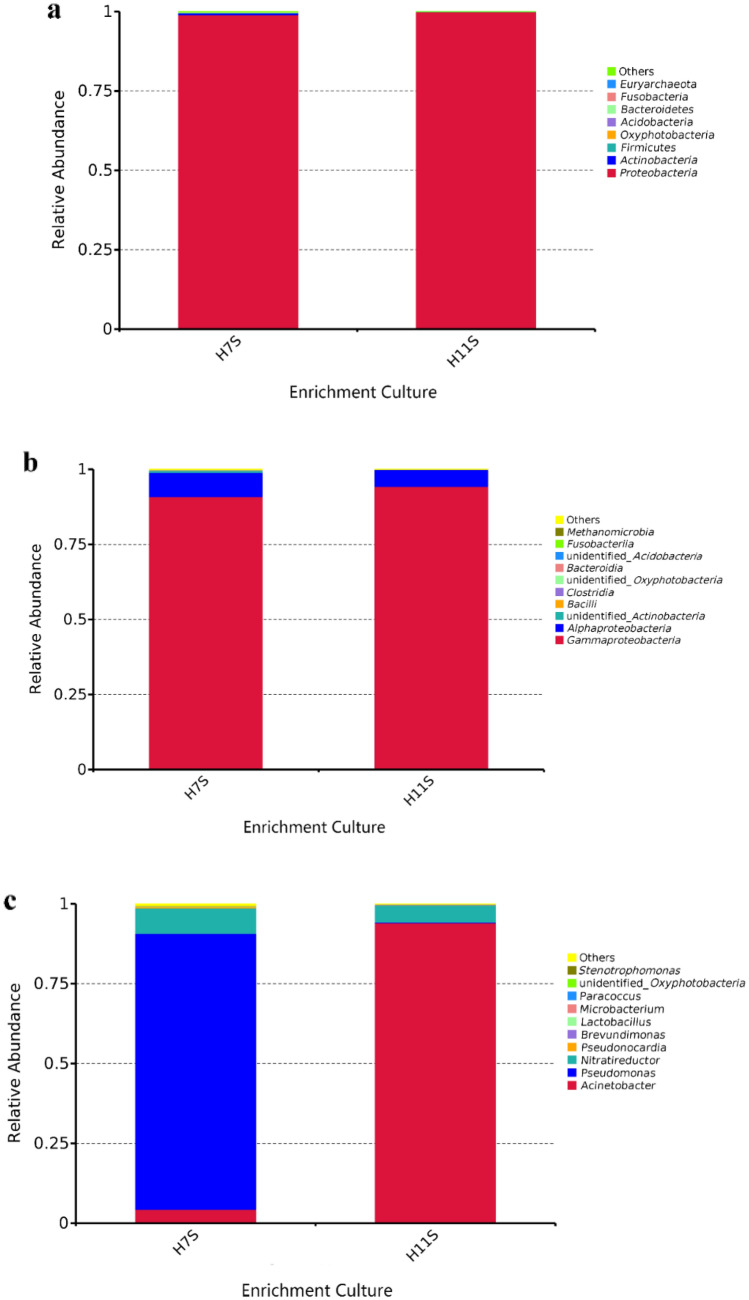
Bacterial community structure at different levels (**a** phylum level. **b** class level. **c** genus level).

In our study, the microbial OTUs of the two enrichment cultures were identified at different levels via phylogenetic analysis and taxonomic distribution. At the phylum and class levels (Fig. 4a,b), the top 10 most abundant species in the two enrichment cultures are listed as a cylindrical accumulation chart. Proteobacteria is the principal group in environments contaminated by hydrocarbons^28,29^, and it also exhibited the highest richness in the two enrichment cultures: 99% and 98% for H7S and H11S, respectively. The abundances of Actinobacteria, Firmicutes, and Bacteroidetes was very low in both enrichment cultures. These phyla have been found to be involved in hydrocarbon biodegradation^30–32^. Actinobacteria not only constitute one of the most important phylogenetic groups in marine ecosystems but also play a leading role in anaerobic TPH degradation-related bioremediation^30,31^. The global biogeography and ecological preferences of Oxyphotobacteria, another common phylum represented by a very low proportion, remain particularly undetected. However, regional studies have shown that Oxyphotobacteria can tolerate high temperature, water stress and ultraviolet radiation^33^. The functioning of Oxyphotobacteria needs further study. As shown in Fig. 4b, Gammaproteobacteria was the major class of Proteobacteria in the H7S and H11S enrichment cultures, accounting for 90.9% and 95.6% of the total OTUs respectively, followed by Alphaproteobacteria (4.3% and 8.0%). Some classes belonging to the phylum Actinobacteria existed only in the enrichment culture H7S, including Acidobacteria and Deltaproteobacteria. On the whole, the two enrichment cultures were similar in community structure at the phylum and class levels.

At the genus level, significant differences between the OTUs of the two enrichment cultures were observed (Fig. 4c). The top 10 genera in each enrichment culture are listed in Fig. 4c. The genera with relatively low abundance were merged into other genera. The enrichment culture H7S was mainly composed of *Pseudomonas* (86.5%), followed by *Nitratireductor* (7.8%) and *Acinetobacter* (4.3%). The enrichment culture H11S was dominated by *Acinetobacter*, whose richness exceeded 95.4%, followed by *Nitratireductor, Brevundimonas*, and *Pseudomonas*, and these results were consistent with the proportion at the phylum and class levels. *Acinetobacter* has the potential to degrade NAP and PHE^34^. *Nitratireductor* shows an excellent oil removal capacity^35^. *Brevundimonas* plays a key role in the in situ bioremediation of oil spills^18^. *Pseudomonas* is commonly used as a model organism for pollutant degradation^36^ because it mediates the degradation of certain organic compounds and anthropogenic contaminants^37,38^. The abundance ratio of the dominant genera *Acinetobacter* : *Nitratireductor* : *Brevundimonas* : *Pseudomonas* was 95:4:0.2:0.2. In Fig. 4c, it is shows that the two enrichment cultures contained small components of *Pseudonocardia*, *Paracoccus* (able to degrade phenanthrene and fluoropolycyclic aromatics in liquid medium^39^) and a biosurfactant-producing bacterium: *Lactobacillus*^40^. Some genera, including *Microbacterium* and *Stenotrophomonas* which are typical oil-degrading bacteria^41^ and efficient biosurfactant-producing bacteria used for the biodegradation of diesel and engine oil^42^, respectively, only existed in the enrichment culture H7S.

According to the abundance information of all samples, the top 35 genera were selected by cluster analysis and displayed in the heat map (Online Resource Fig. S3). The dominant genera of the enrichment cultures H7S and H11S were obviously different. The dominant genera of H11S were *Acinetobacter*, *Brevundimonas*, *Paracoccus* and an unidentified *Clostridiales*. The species clustering results showed that the species of H11S were close to each other. However, the dominant genus was *Pseudomonas* in H7S and the microbial diversity of H7S was higher than that of H11S.

### Isolation and identification of strains

**Figure 5:**
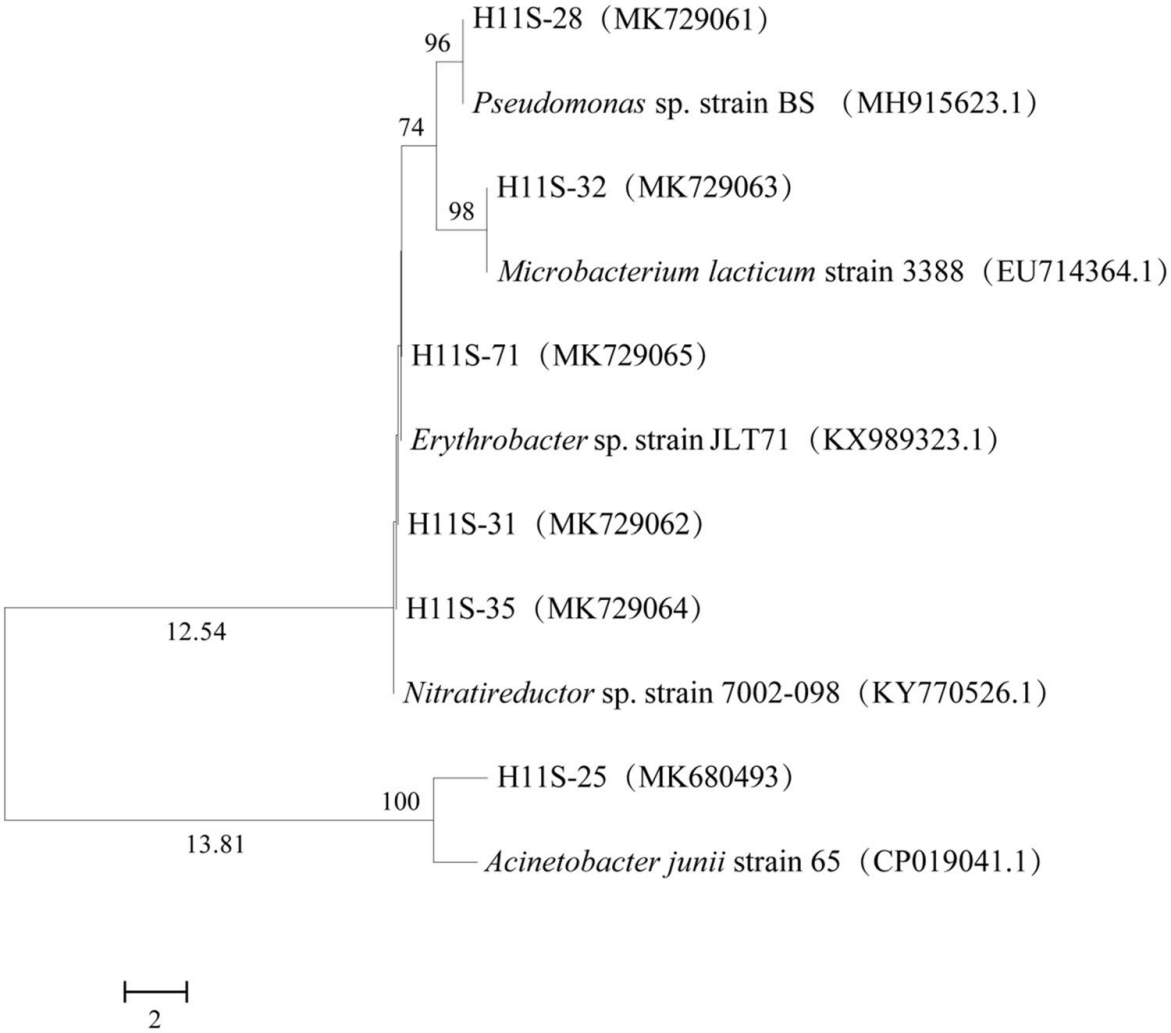
Phylogenetic tree of the oil-degrading strains isolated from the deep-sea sediment samples based on 16S rRNA gene sequences.

The enrichment culture H11S was obtained via approximately two months of enrichment. Six phylogenetically different strains summarized in Table1 have been closely related to a corresponding type strain. The 6 isolated strains showed very close genetic relationships with the corresponding type strain (all above 99%). The bacteria in the enrichment culture H11S belonged to two phyla, *Proteobacteria* and *Actinobacteria*. The main bacterial phylum was *Proteobacteria*, accounting for 80% of the bacterial community H11S. The phylogenetic trees are shown in Fig. 5. The results indicate that the six strains in the enrichment culture H11S belonged to five genera within *Proteobacteria* and *Actinobacteria*. The genus *Nitratireductor* was predominant, and was dominant in the enrichment culture H11S based on the sequencing analysis (Fig. 4c). The 16S rRNA gene sequences of these isolated strains were submitted to the NCBI GenBank database, and the accession numbers are shown in Table 1.

### Biodegradability of crude oil by the isolated strains

Due to ability for oil degradation, the enrichment culture H11S was chosen for the isolation of oil-degrading bacterial strains. Six isolated strains from different genera were selected and their crude oil removal efficiencies were estimated by the gravimetric method (Table 3). The abilities of the six strains to degrade crude oil were calculated by comparison with uninoculated controls. Among the six strains, *Pseudomonas* sp. strain H11S-28 exhibited the best crude oil degradation effects, degrading 44.3% of the crude oil, followed by *Nitratireductor* sp. strain H11S-31 (39.5%) and *Acinetobacter junii* strain H11S-25 (38.5%).

**Table 3:** Degradation efficiencies of crude oil, total *n*-alkane and PAHs by 6 isolated strains from the enrichment culture H11S.

Enrichment culture	Strains	% of crude oil	% of total *n*-alkanes	% of total PAHs
H11S	H11S-25	38.5	29.6	32.4
	H11S-28	44.3	66.9	50.3
	H11S-31	39.5	26.9	37.8
	H11S-32	32.3	15.4	22.2
	H11S-35	35.3	26.2	36.6
	H11S-71	33.5	35.6	20.5

The results of GC–MS analysis showed that most strains were equally capable of degrading total *n*-alkanes (C10–C34) and PAHs (Table 3). It is worth noting that the degradation efficiency of *n*-alkane by the enrichment culture H11S was significantly higher than those for the isolated strains, which may reflect the synergistic effect between bacteria^43, 44^. The ability to degrade target hydrocarbons by each strain is shown in Online Resource Fig. S4. Specifically, most strains degraded more short-chain (C10–C19) and medium-long-chain (C20–C24) *n*-alkanes than long-chain (C25–C34) *n*-alkanes (Online Resource Fig. S4a). Strain H11S-25 showed the highest alkane degradation efficiency of 97.2% for a medium-chain *n*-alkane (C24). In contrast to the results described in “Isolation and identification of strains”, bacterial strains in the enrichment culture H11S had poor ability to degrade PR or PH, with the highest consumption efficiencies only reaching 70.3% and 43.7% respectively.

In terms of the degradation of PAHs (Online Resource Fig. S4b), all the strains tended to degrade monomethyl-substituted DBT, monomethyl-substituted CHR, and dimethyl-substituted CHR more readily than other methyl-substituted DBTs and CHRs. Strains H11S-28, H11S-35, and H11S-31 showed similar degradation characteristics for monomethyl-substituted DBT, exhibiting high degradation efficiencies of 66.6%, 63.9%, and 56.6% respectively. However, there were significant differences in the degradation of naphthalene (NAP)/phenanthrene (PHE). The isolated strains preferred to degrade tetramethyl-substituted NAP/PHE. This result was consistent with the GC–MS analysis, which revealed that the isolated strains including *Acinetobacter junii* strain H11S-25, *Nitratireductor* sp. strain H11S-31 and *Pseudomonas* sp. strain H11S-28 generally exhibited favourable degradation abilities for *n*-alkanes and PAHs.

### Oil-degrading characteristics of consortium H

The results for the oil degradation efficiency of consortium H on days 3, 9, 15 and 21 are shown in Table 4. The degradation efficiency of consortium H reached an amazing 45.4% after 3 days of degradation, and there was no increase over the next 6 days. Finally, the maximum degradation efficiency reached 57.1% on day 21. Therefore, the first 3 days of degradation by consortium H constituted the high-efficiency degradation period. It suggests that each bacterium in consortium H was able to quickly adapt to a stressful environment and showed excellent synergistic degradation. Another period of degradation enhancement extended from day 9 to day 15, after which the degradation efficiency increased slowly.

**Table 4:** Degradation efficiencies of crude oil, total *n*-alkane and PAHs by isolated strains or consortia at different day.

Degradation conditions	Strains or consortia	% of crude oil	% of total *n*-alkanes	% of total PAHs
Degradation by consortium H at 3, 9, 15 and 21 day	H3	45.4	74.2	65.7
	H9	45.6	77.5	64.1
	H15	53.9	86.2	69.6
	H21	57.1	93.3	79.4
Degradation by isolated strains and consortia at 21 day	*Acinetobacter junii* strain H11S-25	46.1	77.9	60.0
	*Pseudomonas* sp. strain H11S-28	50.3	90.7	65.4
	*Nitratireductor* sp. strain H11S-31	50.4	86.1	70.5
	A21	49.5	89.1	71.4
	B21	38.3	78.2	56.2
	C21	48.4	89.9	67.6
	H21	57.1	93.3	79.4
Degradation by isolated strains and consortium H at 3 day	*Acinetobacter junii* strain H11S-25	15.6	5.5	12.9
	*Pseudomonas* sp. strain H11S-28	8.0	2.3	2.5
	*Nitratireductor* sp. strain H11S-31	16.0	2.7	9.2
	H3	45.4	74.2	65.7

The degradation efficiencies of total *n*-alkanes (C10-C34) and PAHs detected by GC–MS showed the same trend as the results detected by the gravimetric method. The degradation efficiency of *n*-alkane was 74.2% on the third day, and reached 86.2% on the 15th day. The highest degradation efficiency was 93.3% after 21 days of degradation. In particular, consortium H exhibited an excellent ability to degrade PAHs. The degradation efficiency for PAHs was 65.7% on the third day, and the final degradation efficiency for PAHs reached 79.4% on the 21st day.

As shown in the GC–MS analysis (Table 5), the degradation characteristics of consortium H for short-chain *n*-alkanes at each time point (days 3, 9, 15 and 21) were almost the same as those determined by the gravimetric method. It was also found that consortium H showed significant degradation of PR on the third day, reaching 84.5%. However, unexpectedly, consortium H did not exhibit an outstanding ability to degrade PH, only reaching 61.6%. Considering the efficiency of PH degradation in the original enrichment culture H11S, it is likely that some bacteria in H11S such as strains of *Erythrobacter*, preferred to degrade PH^45^. In the process of medium-chain and long-chain *n*-alkane degradation, consortium H exhibited the same trend as for the degradation of PAHs. The time of degradation by three-strain consortium increased with the increase of alkane chain length. This phenomenon was consistent with the fact that the degradation efficiency of petroleum hydrocarbons decreased as the carbon number of petroleum compounds increased^46^. Consortium H degraded PAHs mainly included monomethyl-substituted and dimethyl-substituted NAPs (Table 6). The degradation efficiency rapidly increased to 79.7% and 73.2% on the 15th day respectively. The degradation efficiency for other PAHs including high-molecular-weight PAHs increased steadily from 2.7% to 15.6%. In particular, consortium H showed excellent degradation efficiency (above 80%) of CHR on the third day and the degradability remained the same thereafter. This result is also consistent with findings of a previous study showing that consortia were able to exert a stronger positive effect on the biodegradation of PAHs with increasing ring number^27^. In our study, the biodegradation efficiencies for PAHs and high-molecular-weight PAHs were superior to those previously reported of constructed artificial consortia^47^.

**Table 5:** The n-alkanes degradation efficiency in the crude oil after biodegrading 3, 9, 15 and 21 days by consortium H.

Consortia	n-C10	n-C11	n-C12	n-C13	n-C14	n-C15	n-C16	n-C17	PR	n-C18	PH	n-C19	n-C20	n-C21
H3	100.0	56.0	68.5	77.4	93.3	76.1	80.2	78.1	84.5	72.7	53.7	68.8	66.6	68.6
H9	71.6	61.2	71.5	77.0	96.7	79.4	79.3	76.9	91.8	75.0	75.5	73.9	72.6	72.8
H15	100.0	100.0	99.0	99.5	95.2	97.2	95.2	91.9	85.3	87.9	55.4	85.4	82.3	80.8
H21	100.0	100.0	99.7	99.8	95.9	99.4	98.4	90.1	87.0	95.7	61.6	94.9	93.2	93.1

Consortia	n-C22	n-C23	n-C24	n-C25	n-C26	n-C27	n-C28	n-C29	n-C30	n-C31	n-C32	n-C33	n-C34	
H3	68.3	70.2	69.1	69.3	72.0	73.7	73.3	55.3	68.7	− 14.0	74.6	36.0	41.3	
H9	71.6	72.3	72.0	73.3	74.5	75.4	75.7	63.9	76.6	23.3	84.0	59.9	66.6	
H15	78.3	77.7	77.3	78.5	82.3	84.8	83.6	65.9	70.6	− 15.1	73.3	16.4	35.5	
H21	92.0	91.6	91.2	90.4	93.1	94.6	94.3	86.8	88.5	51.1	88.6	64.5	60.6	

n-Cm: alkane with m carbon atom, for instance, n-C16 is alkane with 16 carbon atom; "PR" is pristane, "PH" is phytane.

**Table 6:** The alkylated PAHs degradation efficiency in the crude oil after biodegrading 3, 9, 15 and 21 days by consortium H.

Consortia	C1-NAP	C2-NAP	C3-NAP	C4-NAP	C1-Flu	C2-Flu	C3-Flu	C1-PHE	C2-PHE
H3	58.2	60.9	61.6	64.0	70.8	72.2	74.9	63.7	62.0
H9	58.7	58.1	62.1	59.6	68.4	68.1	72.7	64.7	57.9
H15	79.7	73.2	66.6	65.2	71.1	72.3	75.5	66.5	61.9
H21	86.3	97.1	75.2	69.5	78.5	84.6	82.1	75.9	72.3

Consortia	C3-PHE	C4-PHE	C1-DBT	C2-DBT	C3-DBT	C1-CHR	C2-CHR	C3-CHR	
H3	60.2	68.0	80.5	76.5	75.1	74.6	81.8	85.1	
H9	58.0	67.1	80.4	76.5	68.9	77.9	83.8	88.7	
H15	61.2	70.9	84.5	82.2	79.1	77.8	83.1	89.3	
H21	75.8	78.8	88.8	86.2	85.5	84.1	88.8	87.8	

NAP, FLU, DBT, PHE, and CHR represent for naphthalene, fluorine, dibenzothiophene, phenanthrene, chrysene, respectively. C1(C2, C3, C4)-NAP, alkylated naphthalene with straight-chain of 1 ~ 4 carbon atoms; C1(C2, C3)-FLU, alkylated fluorene with straight-chain of 1 ~ 3 carbon atoms; C1(C2, C3)-DBT, alkylated dibenantherene with straight-chain of 1 ~ 3 carbon atoms; C1(C2, C3, C4)-PHE, alkylated phenanthrene with straight-chain of 1 ~ 4 carbon atoms; C1(C2)-CHR, alkylated chrysene with straight-chain of 1 ~ 2 carbon atoms).

GC–MS analysis showed that consortium H degraded most of the total *n*-alkanes and total PAHs in oil on the third day. The total oil degradation efficiency calculated by gravimetric analysis indicated that the efficiency of oil removal by consortium H could exceed 50% after 15 days of degradation.

### Biodegradability of crude oil by the isolated strains and consortia after degradation for 21 days

After 21 days of degradation, the various crude oil degradation characteristics of the isolated strains and consortia were compared. The degradation efficiency of consortium H, which was composed of three different bacterial species, was higher than those for isolated strains and other consortia. The degradation efficiencies of consortia A21, B21 and C21 were not as high as that for *Nitratireductor* strain H11S-31, which may be due to competition or antagonistic effects between the two artificially combined bacterial strains. Compared with the degradation efficiency of consortium H, *Acinetobacter junii* strain H11S-25 showed lower degradation efficiency (46.1%) but played a key role in the overall degradation system. This strain probably relieved the competition or antagonism between *Pseudomonas* sp. strain H11S-28 and *Nitratireductor* sp. H11S-31.

In addition, GC–MS was used to measure the degradation efficiencies for total alkanes and PAHs. Total alkane and PAHs degradation efficiencies of consortium H reached the highest values of 93.3% and 79.4% respectively. Compared with the corresponding constituent strains, the removal efficiency for total PAHs of consortium A21 improved from 0.9% to 11.4% (Table 4), indicating that strain H11S-25 and strain H11S-31 exerted mutually promoting effects. Perhaps the metabolites produced by the two strains promoted the growth of each other to increase their usage of oil. However, the low total alkane and PAHs degradation efficiencies of consortium B21 (78.2% and 56.2%, respectively) indicated that strain H11S-31 and strain H11S-28 exhibited obvious degradation inhibition effects. The possible reason is that the strains inhibited each other metabolic capacities via producing extracellular substances^47^. The degradation ability for total PAHs of consortium C21 indicated that there was a weak degradation-promoting effect between strain H11S-25 and strain H11S-28.

**Figure 6:**
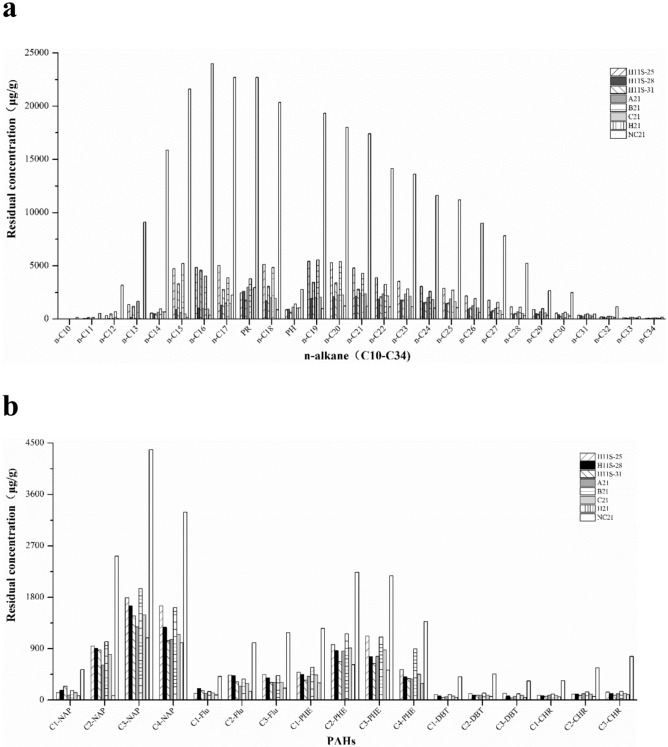
The n-alkanes and PAHs residual concentration in the crude oil after biodegrading 21 days by isolated strains and consortia (**a** n-Cm: alkane with m carbon atom, for instance, n-C16 is alkane with 16 carbon atom; "PR" is pristane, "PH" is phytane. **b** NAP, FLU, DBT, PHE, and CHR represent for naphthalene, fluorine, dibenzothiophene, phenanthrene, chrysene, respectively. C1(C2, C3, C4)-NAP, alkylated naphthalene with straight-chain of 1 ~ 4 carbon atoms; C1(C2, C3)-FLU, alkylated fluorene with straight-chain of 1 ~ 3 carbon atoms; C1(C2, C3)-DBT, alkylated dibenantherene with straight-chain of 1 ~ 3 carbon atoms; C1(C2, C3, C4)-PHE, alkylated phenanthrene with straight-chain of 1 ~ 4 carbon atoms; C1(C2)-CHR, alkylated chrysene with straight-chain of 1 ~ 2 carbon atoms).

As shown by the GC–MS analysis (Fig. 6a), consortium A21 showed superior activity (above 89.6%) in degrading short-chain *n*-alkanes, and this degradation ability was much higher than the performance of strain H11S-25 and strain H11S-31. However, the ability of consortium A21 to degrade PR and PH was unsatisfactory and was lower than that of its constituent strains. The short-chain and medium-chain *n*-alkane degradation efficiencies of consortium B21 were significantly lower than those of the corresponding individual strains. In addition, the short-chain *n*-alkane degradation efficiency of consortium C21 was only slightly better than that of strain H11S-28. However, consortium H showed excellent biodegradability for both short- and medium-chain *n*-alkanes.

The PAHs degradation efficiencies in crude oil are shown in Fig. 6b. Degradation efficiencies for high-molecular-weight PAHs (PHE, DBT, and CHR) of consortia A21, B21, and C21 were not as high as those of the constituent strains. However, the PAHs degradation efficiencies of consortium H exceeded 80.0%, especially for DBT and CHR. That is, with increasing ring number of PAHs, consortium H exhibited a strong positive effect on the biodegradation of those compounds. Our results showed that consortium H had a wider range of degrading capacities for petroleum components. This indicates that the key to constructing an efficient artificial consortium is that the proportion of bacterial strains should strictly follow the proportion of the original enrichment culture.

## Conclusion

In this study, two oil-degrading enrichment cultures were obtained and six oil-degrading strains were screened from the deep-sea hydrothermal region of the South Mid-Atlantic Ridge. The biodegradability of the enrichment cultures and isolated strains was analysed. The results showed that the enrichment culture H11S possessed a higher ability to biodegrade alkanes and PAHs than the enrichment culture H7S. According to the stable bacterial diversity ratio in the enrichment culture H11S, we constructed an artificial consortium H, which had the characteristics of high efficiency and wide range of oil degradation. Our study first demonstrated that constructing an oil-degrading consortium with minimum complexity based on the ratio of bacterial abundance in the original enrichment culture is an effective way to improve oil degradability. Consortium H, which we constructed is expected to be applicable in the bioremediation of petroleum hydrocarbon pollutants for environmental or commercial purposes.

## Supplementary Information

Supplementary Information.
