# Supplementary material for: Reconstruction and evaluation of oil-degrading consortia isolated from sediments of hydrothermal vents in the South Mid-Atlantic Ridge
Source: Sci Rep. 2021 Jan 14;11:1456. doi: 10.1038/s41598-021-80991-5 (PMC7809451; doi:10.1038/s41598-021-80991-5)
Supplement: Supplementary file 1 — Supplementary Information. [file 41598_2021_80991_MOESM1_ESM.docx]

***Supplementary Material***

Reconstruction and evaluation of oil-degrading consortia isolated from sediments of hydrothermal vents in the South Mid-Atlantic Ridge

Meng Ma^1, 2, a^, Li Zheng^2, 3, a*^, Xiaofei Yin^2^, Wei Gao^2^, Bin Han^2^, Qian Li^2^, Aimei Zhu^2^, Hao Chen^2^, Huanghao Yang^1*^

^a^ These authors contributed equally to this work.

1 College of Biological Science and Engineering, Fuzhou University, Fuzhou 350108, China

2 Key Laboratory of Marine Eco-Environmental Science and Technology, First Institute of Oceanography, Ministry of Natural Resources, Qingdao, 266061, China

3 Laboratory for Marine Ecology and Environmental Science, Pilot National Laboratory for Marine Science and Technology (Qingdao), Qingdao, 266071, China

^*^ Corresponding author: Li Zheng: [zhengli@fio.org.cn](mailto:zhengli@fio.org.cn) ; Huanghao Yang: [hhyang@fzu.edu.cn](mailto:hhyang@fzu.edu.cn)

**Supplementary Table S1** The ambient environmental parameters of sediment samples

| **Sample** | **Sites** | | **Depth**  **( m )** | **Ambient Temperature**  **( °C )** | **Salinity**  **( PSU )** |
| --- | --- | --- | --- | --- | --- |
| 7S | 27°08ˊS | 13°28ˊW | 3400 | 2.59 | 34.87 |
| 11S | 26°00ˊS | 13°51ˊW | 2594 | 2.56 | 34.86 |


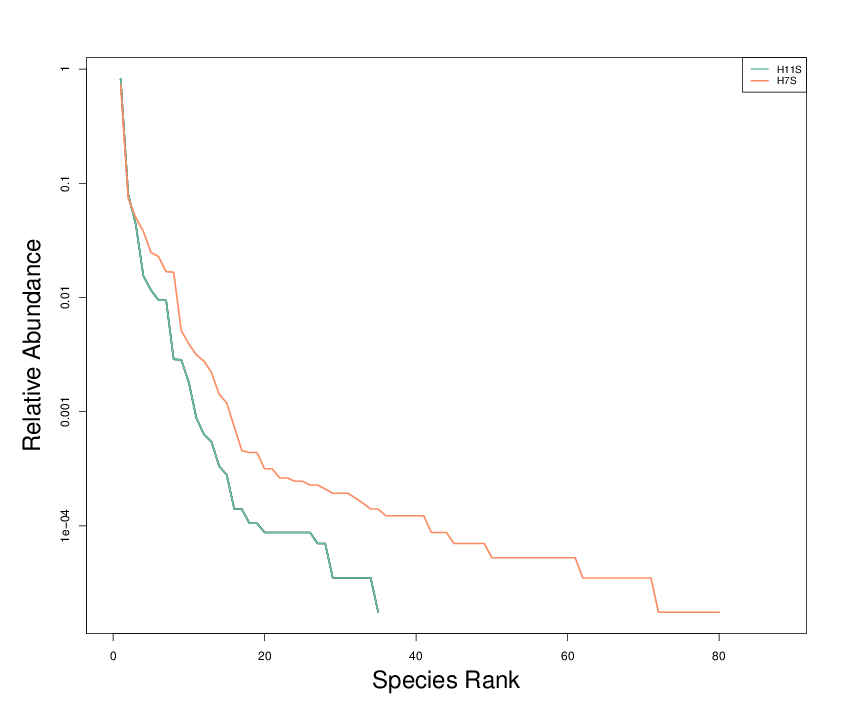


**Supplementary Fig. S1** Rank Abundance curve: the abscissa is the ordinal number sorted by OTUs abundance, and the ordinate is the relative abundance of corresponding OTUs.


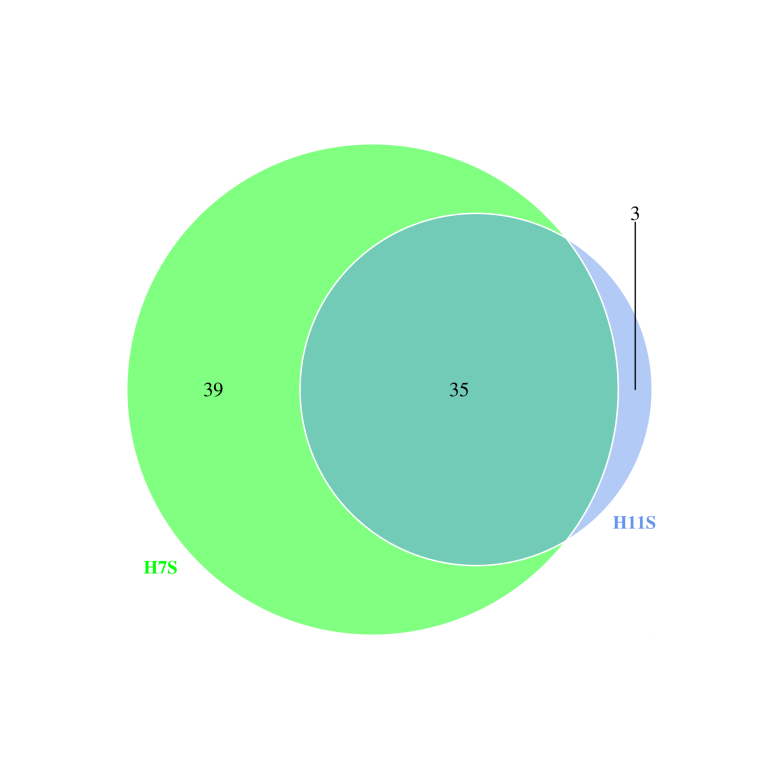


**Supplementary Fig S2** Venn diagram of the enrichment cultures H7S and H11S shows that 35 OTUs are shared. Venn diagram of unique and shared OTUs, the numbers of OTU are presented separately on both sides represent unique OTU of each library.


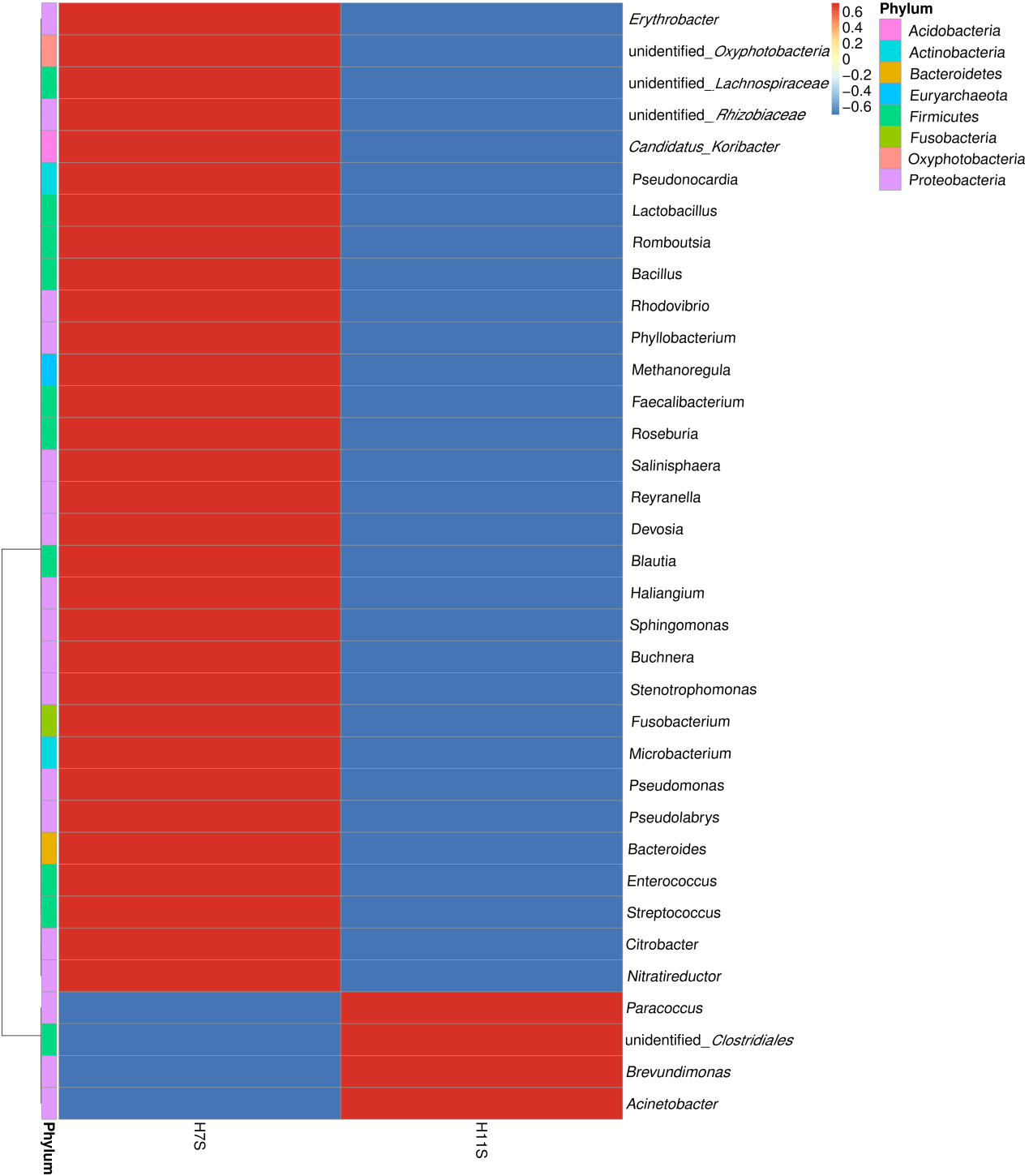


**Supplementary Fig S3** The heat map of the top 35 genera of the enrichment cultures by cluster analysis.

**a**


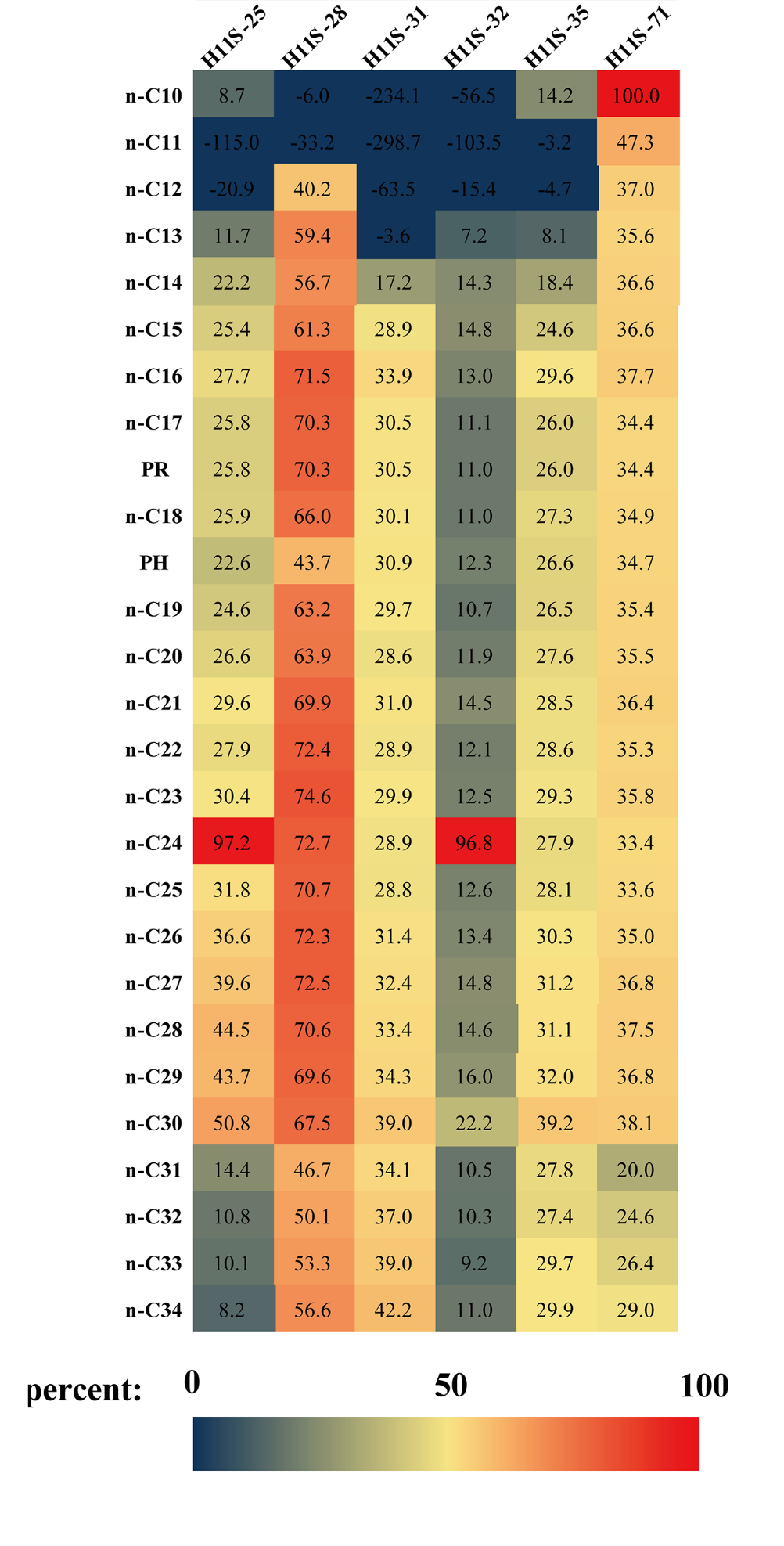


**b**

**
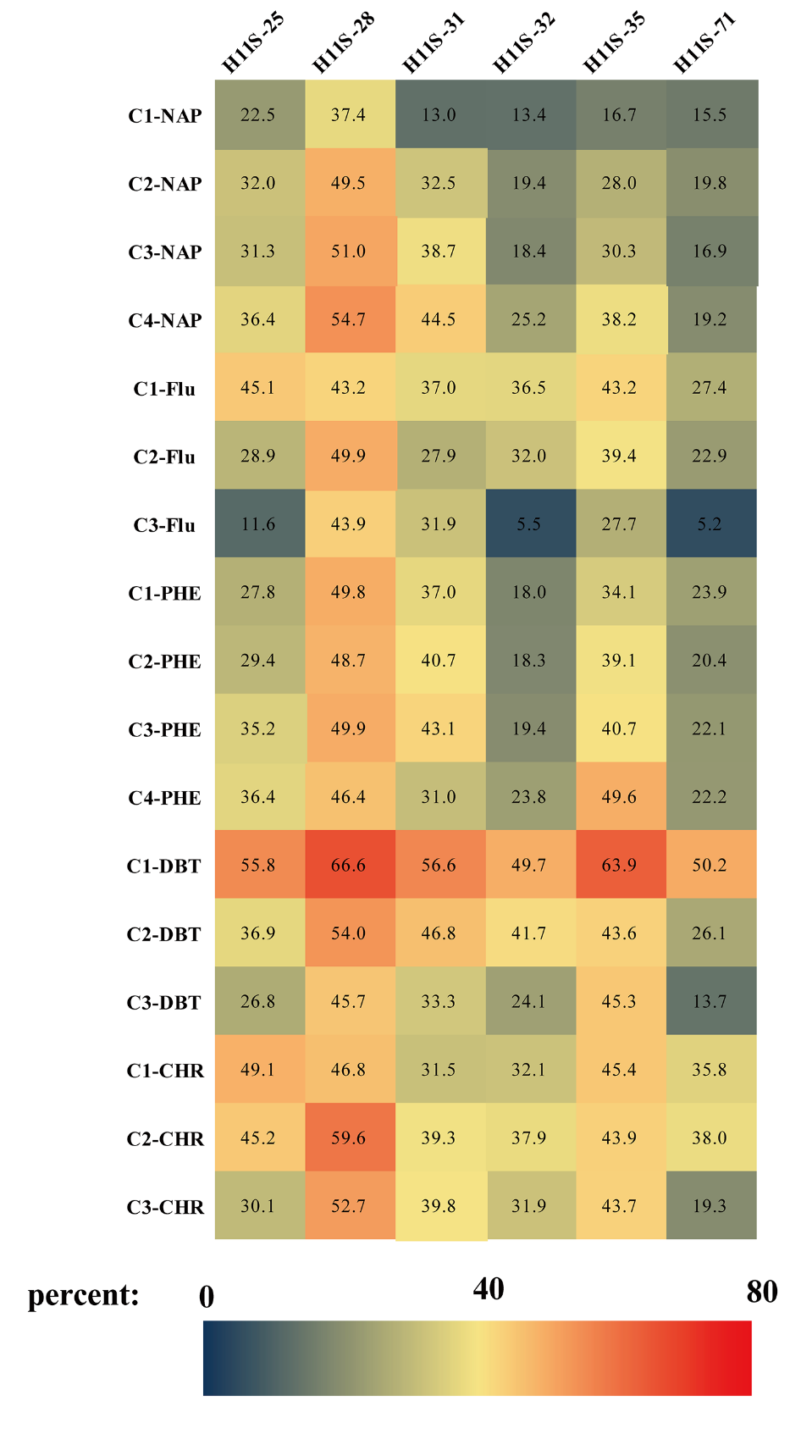
**

**Supplementary Fig S4** Heat map of the degradation efficiency of n-alkanes and PAHs by 6 isolated strains from the enrichment culture H11S (**a** n-Cm: alkane with m carbon atom，for instance，n-C16 is alkane with 16 carbon atom; "PR" is pristine, "Ph" is phytane. **b** NAP, FLU, DBT, PHE, and CHR represent for naphthalene, fluorine, dibenzothiophene, phenanthrene, chrysene, respectively. C1(C2, C3, C4) –NAP, alkylated naphthalene with straight-chain of 1-4 carbon atoms; C1(C2, C3) –FLU, alkylated fluorene with straight-chain of 1 ~ 3 carbon atoms; C1(C2, C3) –DBT, alkylated dibenantherene with straight-chain of 1 ~ 3 carbon atoms; C1(C2, C3, C4) –PHE, alkylated phenanthrene with straight-chain of 1 ~ 4 carbon atoms; C1(C2)-CHR, alkylated chrysene with straight-chain of 1 ~ 2 carbon atoms).
